# Comparison of three video head impulse test systems for the diagnosis of bilateral vestibulopathy

**DOI:** 10.1007/s00415-020-10060-w

**Published:** 2020-07-27

**Authors:** T. S. van Dooren, D. Starkov, F. M. P. Lucieer, B. Vermorken, A. M. L. Janssen, N. Guinand, A. Pérez-Fornos, V. Van Rompaey, H. Kingma, R. van de Berg

**Affiliations:** 1 Division of Balance Disorders, Department of Otorhinolaryngology and Head and Neck Surgery, Maastricht University Medical Centre, Maastricht, The Netherlands; 2 Faculty of Physics, Tomsk State Research University, Tomsk, Russia; 3 Faculty of Health, Medicine and Life Sciences, University of Maastricht, Maastricht, The Netherlands; 4 Department of ENT/Audiology, School for Mental Health and Neuroscience (MHENS), Maastricht University Medical Centre, Maastricht, The Netherlands; 5 Department of Methodology and Statistics, Care and Public Health Research Institute (CAPHRI), Maastricht University, Maastricht, The Netherlands; 6 Service of Otorhinolaryngology Head and Neck Surgery, Department of Clinical Neurosciences, Geneva University Hospitals, Geneva, Switzerland; 7 Faculty of Medicine and Health Sciences, University of Antwerp, Antwerp, Belgium; 8 Department of Otorhinolaryngology and Head and Neck Surgery, Antwerp University Hospital, Edegem, Belgium

**Keywords:** VHIT, Video head impulse test, VOR gain, BV, Bilateral vestibulopathy, Covert saccades

## Abstract

**Introduction:**

A horizontal vestibulo-ocular reflex gain (VOR gain) of < 0.6, measured by the video head impulse test (VHIT), is one of the diagnostic criteria for bilateral vestibulopathy (BV) according to the Báràny Society. Several VHIT systems are commercially available, each with different techniques of tracking head and eye movements and different methods of gain calculation. This study compared three different VHIT systems in patients diagnosed with BV.

**Methods:**

This study comprised 46 BV patients (diagnosed according to the Báràny criteria), tested with three commercial VHIT systems (Interacoustics, Otometrics and Synapsys) in random order. Main outcome parameter was VOR gain as calculated by the system, and the agreement on BV diagnosis (VOR gain < 0.6) between the VHIT systems. Peak head velocities, the order effect and covert saccades were analysed separately, to determine whether these parameters could have influenced differences in outcome between VHIT systems.

**Results:**

VOR gain in the Synapsys system differed significantly from VOR gain in the other two systems [*F*(1.256, 33.916) = 35.681, *p* < 0.000]. The VHIT systems agreed in 83% of the patients on the BV diagnosis. Peak head velocities, the order effect and covert saccades were not likely to have influenced the above mentioned results.

**Conclusion:**

To conclude, using different VHIT systems in the same BV patient can lead to clinically significant differences in VOR gain, when using a cut-off value of 0.6. This might hinder proper diagnosis of BV patients. It would, therefore, be preferred that VHIT systems are standardised regarding eye and head tracking methods, and VOR gain calculation algorithms. Until then, it is advised to not only take the VOR gain in consideration when assessing a VHIT trial, but also look at the raw traces and the compensatory saccades.

## Introduction

Bilateral vestibulopathy (BV) is a heterogeneous chronic condition in which the vestibular function is severely impaired or absent in both ears [1]. A greatly reduced or absent vestibulo-ocular reflex (VOR) is a main clinical marker of BV, among other symptoms [2]. To quantify the VOR function in all planes of the semicircular canals, the video head impulse test (VHIT) is widely used [3]. The vestibulo-ocular reflex gain (VOR gain) is considered to be the main outcome parameter of the VHIT. VOR gain represents the relationship between eye and head velocity, and can be calculated in various ways. For example, VOR gain can be calculated as the ratio between eye and head velocity at a certain point in time, at peak head velocity, or throughout the whole head movement (i.e. the area under the curve gain, regression analysis) [4–6]. VOR gain should be close to 1.0 in healthy subjects [7]. Therefore, a decreased VOR function should result in a decreased VOR gain. Moreover, a horizontal angular VOR gain of < 0.6 on both sides, as measured by the VHIT, is one of the diagnostic criteria for BV according to the Bárány Society [8].

BV patients can also show catch-up saccades during the VHIT. These saccades are a compensation mechanism for the retinal slip during head movements, and can occur during or shortly after a head impulse (“covert” saccades and “overt” saccades, respectively). As an adaptation effect, the latency of the catch-up saccades can decrease and therefore, the amount of covert saccades can increase [9]. These covert saccades could influence VOR gain calculations, especially when area under the curve gain calculation is used.

Several VHIT systems are commercially available, each with different methods of gain calculation and different techniques of tracking eye and head movements. Small study populations show significant differences in VOR gain between different VHIT systems within healthy subjects and patients. Despite these differences in VOR gain, all systems identified vestibular deficits similarly [5, 6]. It is unknown what the effect of using different VHIT systems is on the VOR gain in subjects with severely impaired vestibular function on both ears. In case the use of different VHIT systems would result in different clinical diagnoses within the same patient (e.g. classifying a patient “yes” or “no” with BV), it might be necessary to standardise systems regarding VOR gain calculation algorithms and eye and head tracking methods.

Objective of this study was to compare three commercial VHIT systems (Interacoustics, Otometrics, and Synapsys) in a large group of BV patients. Main outcome parameters were horizontal VOR gain as calculated by the system, and the agreement between the systems on identifying BV according to the diagnostic criteria (horizontal VOR gain < 0.6). Since there are technological differences inherent to the VHIT systems (i.e. different VOR gain calculation algorithms and different head and eye tracking), it was hypothesised that different VHIT systems could lead to clinically relevant differences in VHIT outcome within the same BV patient.

## Methods

### Study population

This study comprised 46 patients diagnosed with BV at the Division of Balance Disorders at Maastricht University Hospital, based on the diagnostic criteria for BV from the Bárány Society [8]. Since VOR gain obtained by VHIT was used as an outcome parameter in this study, this criterium was removed from the inclusion criteria. Patients diagnosed with BV solely based on VHIT outcomes were, therefore, not part of this study population. Inclusion criteria comprised (1) reduced caloric response (sum of bithermal maximum peak slow phase eye velocities of < 6°/s on each side), (2) and/or reduced horizontal angular VOR gain < 0.1 on rotatory chair and a phase lead > 68°. Exclusion criteria comprised being unable to stop vestibular suppressants for 1 week (cinnarizine and all psychiatric medication), and the inability to undergo one of the vestibular examinations.

### Testing protocol

#### Experimental setup [7]

One trained examiner (FL) performed all VHIT’s. A fixed distance of two metres from the back of the chair to the point of fixation was ensured [10]. Patients were seated on a static chair, to prevent upper body movement during head impulses. The room was well lit, to ensure a small pupil in every patient. Patients fixated on a green (532-nm) 1-mw laser dot projected on a large full visual field black (or white) painted wall. This facilitated a wider range for measuring the eye movements. At the same time, it minimised the change of artefacts due to light reflections onto the pupil. The fixating point was adjusted to the eye level of every patient. Each test started with calibration of the system. The examiner assessed the quality of the calibration and determined whether the process needed to be repeated. The examiner stood behind the patient, holding the head firmly during head impulses. Patients were instructed to relax their neck, keep their eyes wide open and fixate on the target in front of them. The examiner continuously repeated these instructions to facilitate optimal awareness of the patient. The head impulses comprised fast horizontal rotational head movements (> 120°/s) with a low amplitude, unpredictable in timing and direction. Only outward impulses were given [11].

**Fig. 1:**
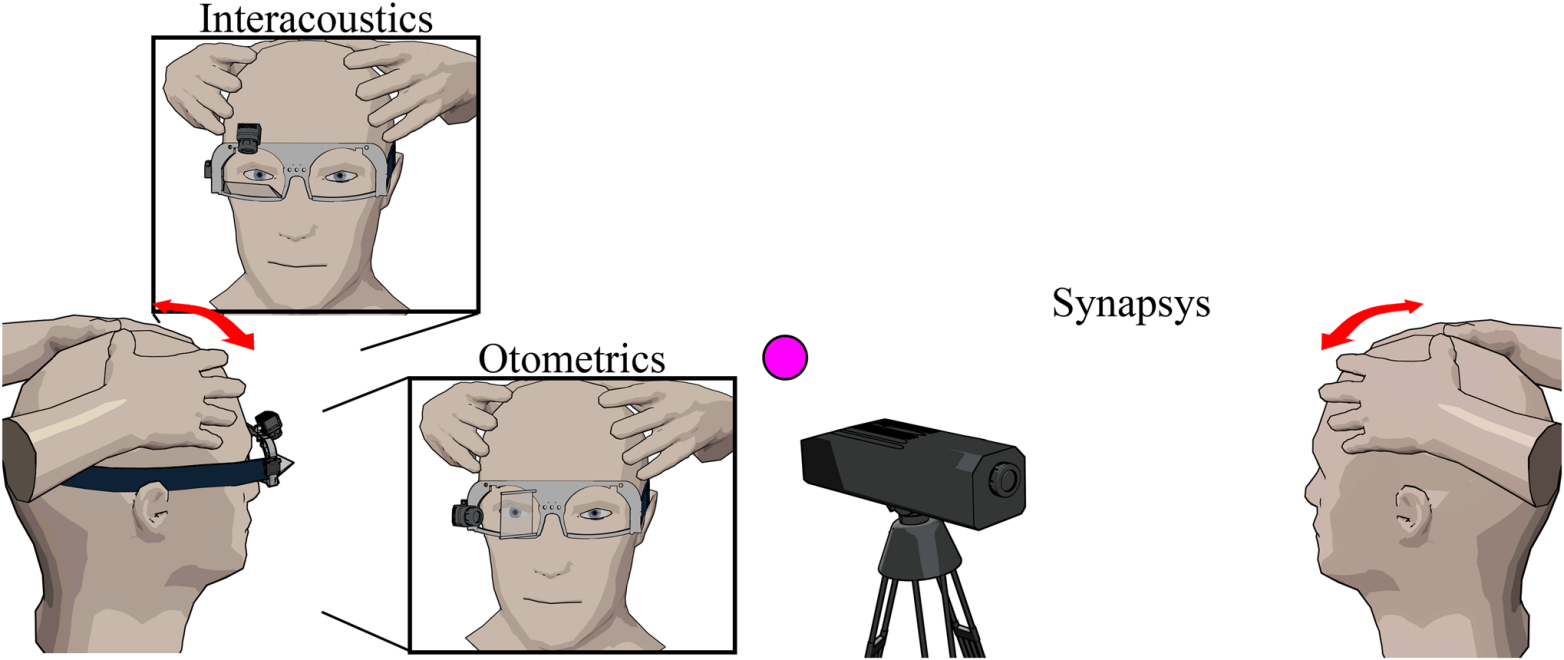
Animations of the three VHIT systems used in this study. The Interacoustics and Otometrics VHIT systems both consist of a pair of goggles with a build-in eye and head movement tracking system. The Synapsys VHIT system comprises a space-fixed camera placed in front of the patient

The camera of the Interacoustics and Otometrics systems is head fixed and is integrated in a pair of goggles. Therefore, before start of testing, goggle movement was minimised by tightly fastening the strap of the goggles around the patients’ head. The camera was always set on the right eye and focused on the pupil while the patient looked at the point of fixation with eyes wide open. In case the eyelids were in front of the pupil, the examiner adjusted the rim of the goggles so they would hold the eyelids back. After calibration, the patient was instructed to not touch (the strap of) the goggles, their face and/or their hair. The camera of the Synapsys system is space fixed, and therefore, no goggles were used. The camera that measured eye and head movements was placed in front of the patient. Eye movements from both eyes were measured (Fig. 1).

#### VHIT systems

Three different VHIT systems were used in this study: EyeSeeCam (Interacoustics VOG; Munich, Germany), ICS Impulse (GN Otometrics; Taastrup, Denmark), and Ulmer (Synapsys, Marseille, France). Each patient sequentially underwent the horizontal VHIT with the different VHIT systems. The Synapsys system was not used in 17 patients, and the Interacoustics system was not used in one patient, due to the unavailability of the systems at the time of testing. The order of testing of the different VHIT systems was randomised by draw.

### VOR gain calculation by the different VHIT systems

VOR gain, as calculated by the systems, was used as main outcome parameter. The three systems calculate VOR gain differently. Interacoustics uses instantaneous gain; it divides eye- and head velocity at a certain point in time (small window around 60 ms) after onset of the head movement [12]. Otometrics calculates VOR gain as the ratio of the area under the eye velocity and head velocity curve (from 60 ms before peak head acceleration to the last value of 0°/s as the head returns to rest). If needed, the eye movement is desaccaded by the system before the VOR gain is calculated [13]. The Synapsys system calculates the VOR gain over the period from 40 ms before to 80 ms after peak head acceleration for each impulse. In case of a covert saccade, the 80-ms window is reduced, and stops at time of onset of the covert saccade [14]. However, the method of gain calculation used by the Synapsys system was unknown to this research group, despite multiple efforts to obtain more information from the manufacturer.

### Covert saccades

Covert saccades might influence VOR gain (calculation). Therefore, covert saccades in this study population were analysed separately to assess whether they differed between tests (as an adaptation effect) in this BV population when repeatedly tested. The frequency of occurrence of covert saccades, and the latency of the first covert saccade of a trace were analysed.

#### Extracting data

To extract saccades, head and eye velocity (Interacoustics and Otometrics) and position (Synapsys) traces were exported and processed using Wolfram Mathematica 11.3 (Wolfram Research, Champaign, IL, USA). Only traces that were accepted by the systems were exported.

#### Pre-processing data

Synapsys measures both eyes during VHIT, but in this study, it was chosen to only use traces from the right eye, to better facilitate comparison with Interacoustics and Otometrics, which only register data from the right eye. In case of missing values from the right eye, data from the left eye were used. Because of the lower resolution of the Synapsys camera (100 Hz), the original eye and head position data were resampled to 250 Hz using linear interpolation. By differentiating these eye and head position traces, the velocity traces were calculated for eye and head movements recorded with the Synapsys system. Eye and head velocity traces from Interacoustics and Otometrics were directly extracted from the system itself. Eye and head position data for these two systems were calculated using numerical integration. Head and eye acceleration data were calculated for all three systems by differentiating the eye and head velocity signal.

#### Cleaning data

To establish artefact-free traces for analysis, traces were removed when (1) peak head velocity was < 120°/s, or (2) the head velocity trace contained a bounce at the end of the impulse of > 50% of peak head velocity, or (3) head velocity never crossed zero after peak head velocity (within the recorded time frame), or (4) the head velocity trace contained missing values, or (5) the shape of the head velocity trace implied an inadequate head impulse, assessed by visual inspection and consensus between three authors (RB, DS, TD), or (6) when the mean head velocity of the interval of 80 ms prior and 120 ms after a peak head velocity was not in the range of ± 3 SD of the set of mean head velocities calculated in the same interval in all traces of one patient [4, 15, 16].

#### Saccade detection

A custom-made algorithm was developed in Mathematica, and applied to extract saccades from the eye traces. To increase accuracy, every saccade was verified by visual inspection in the eye and head velocity and position traces. Two authors needed to achieve consensus (TD, DS) before a saccade was approved. Head impulse onset was specified as head velocity exceeding 10°/s, head impulse offset was defined as head velocity crossing 0°/s. Onset of a saccade was marked as the point where eye velocity crossed 0°/s or eye acceleration reached 2000°/s^2^. Saccades were included when (1) they occurred after peak head velocity, and (2) had a magnitude of more than 60°/s, and (3) peak velocity of the saccade was recorded, and (4) occurred at least in two traces around the same location within the same trial and patient. A saccade was classified as covert when onset occurred before head velocity crossed zero, and as overt when onset occurred after head velocity crossed zero.

#### Saccade analysis: defining frequency and latency

In this study, the first covert saccades of the first seven artefact-free traces were used for analysis [17]. The frequency and latency of the covert saccades were extracted from the original eye velocities in the Interacoustics and Otometrics system, and from the calculated eye velocities in the Synapsys system. The frequency of occurrence of a covert saccade was first registered as a binary outcome (Yes/No) for every trace separately. From these data, a ratio per patient was calculated (in percentage). Latency (in milliseconds) was registered as the onset of the covert saccade, and was normalised to the start of the head impulse [18].

### Statistical analysis

Data were analysed using SPSS Statistics 24 for Windows and R (v.3.5.2.). The α-value was set on *p* < 0.05. In case of multiple comparisons, the Bonferroni correction was applied. When no interaction was found between leftwards and rightwards head impulses, the direction of the impulse was removed from the statistical model and both sides were analysed together.

#### Statistical analysis of VOR gain and agreement of VHIT systems regarding BV diagnosis

A repeated-measures ANOVA was used to compare mean VOR gain between the three systems. A VOR gain of < 0.6 was classified as “bilateral vestibulopathy”, a VOR gain of ≥ 0.6 was classified as “no bilateral vestibulopathy” [8]. In case the VHIT systems showed a discrepancy in classifying BV, it was classified as “no agreement”.

#### Statistical analysis of VOR gain and repetitive testing (the order effect)

To evaluate the order effect, a repeated-measures ANOVA was used to compare mean VOR gain between the first and the last executed VHIT trial (regardless of the VHIT system).

#### Statistical analysis of peak head velocity

Peak head velocities (extracted from the raw traces of the VHIT systems) of all traces of all patients were combined per VHIT system. Median peak head velocities were compared between VHIT systems using a Mann–Whitney *U* test. In patients with “no agreement” between systems, peak head velocities were analysed separately within the BV patient. Median peak head velocities of those particular trials were compared between VHIT systems using a Mann–Whitney *U* test.

#### Statistical analysis of saccades

The frequency of occurrence of covert saccades was compared between the first and the last executed VHIT trial (regardless of the VHIT system) using a generalised linear mixed-effects model. Additionally, the latency of the first covert saccade was compared between the first and the last executed VHIT trial (regardless of the VHIT system) with a paired *T* test. Patients with missing values (no saccades) were not included in this last analysis.

## Results

### Patient characteristics

In total, 46 BV patients were included: 23 males and 23 females. Mean age was 59 years (standard deviation 11 years). Definite and probable etiologies comprised: ototoxic effects of antibiotics (*n* = 8) or chemotherapy (*n* = 1), post-infectious due to Lyme disease (*n* = 1), Hashimoto’s thyroiditis (*n* = 1), Herpes infection (*n* = 1), meningitis (*n* = 2), inherited, e.g. by DFNA9 gene mutation (*n* = 7), bilateral Menière’s disease (*n* = 3), autoimmune disease (*n* = 1). In 21 patients, no etiology could be determined (idiopathic).

**Fig. 2:**
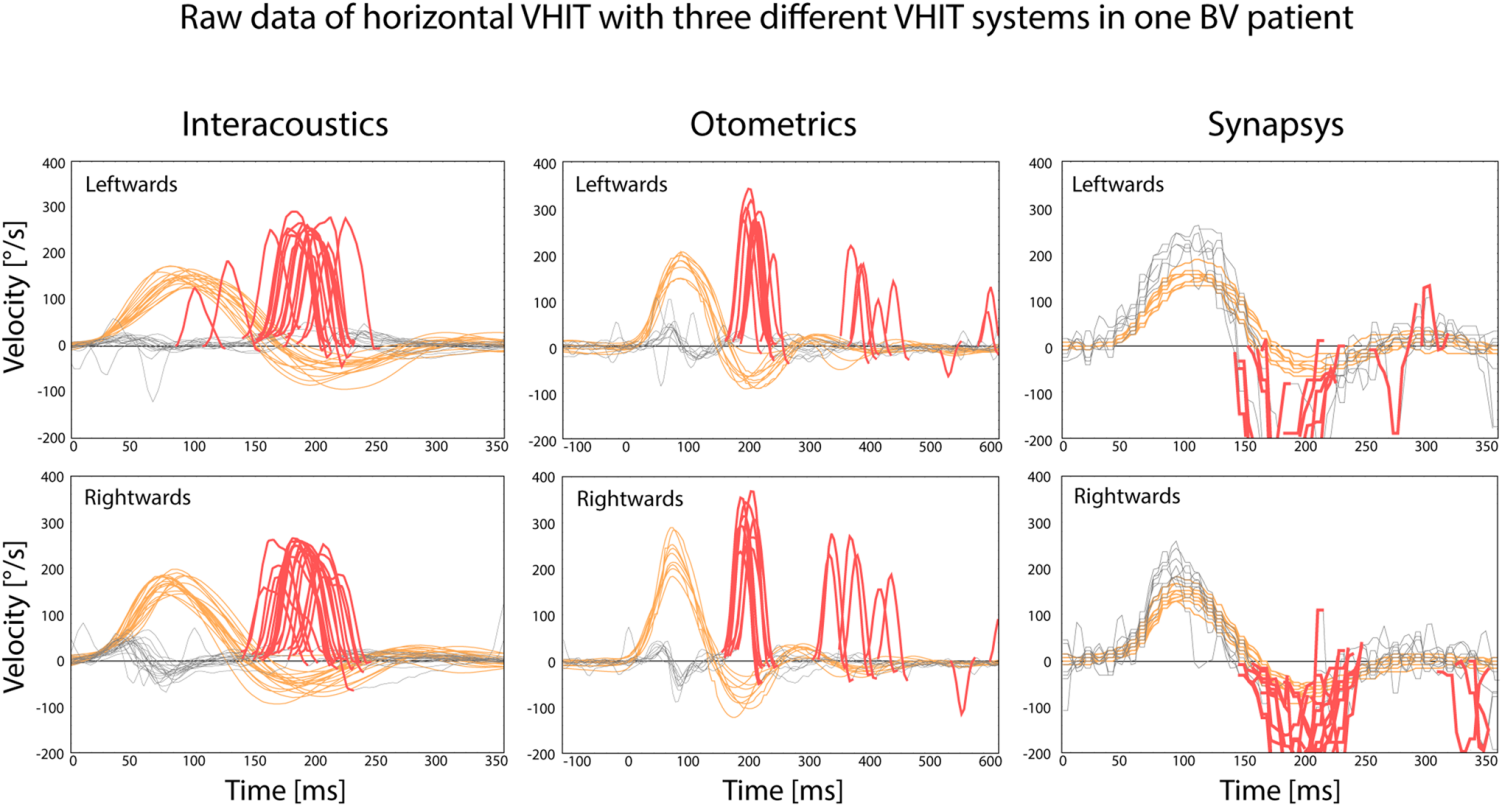
Raw eye and head movement data of one BV patient (patient 21), obtained by three different VHIT systems during three consecutive VHIT trials. Grey dotted lines represent eye movements, orange lines represent head movements, red lines represent saccades. Note that eye movements obtained with the Synapsys system have a different graphical representation. This is based on the fact that a space-fixed camera with a lower sampling rate was used, instead of a head-fixed camera

All three VHIT systems were able to capture the same type of eye movement responses to head impulses. This is illustrated in Fig. 2, which presents the raw data of one BV patient (patient 21), selected as a representative sample of the whole study population. Further details of VHIT characteristics (VOR gain, peak head velocity, timing of saccades) of all tested patients will be discussed below.

### VOR gain and agreement of VHIT systems regarding BV diagnosis

**Fig. 3:**
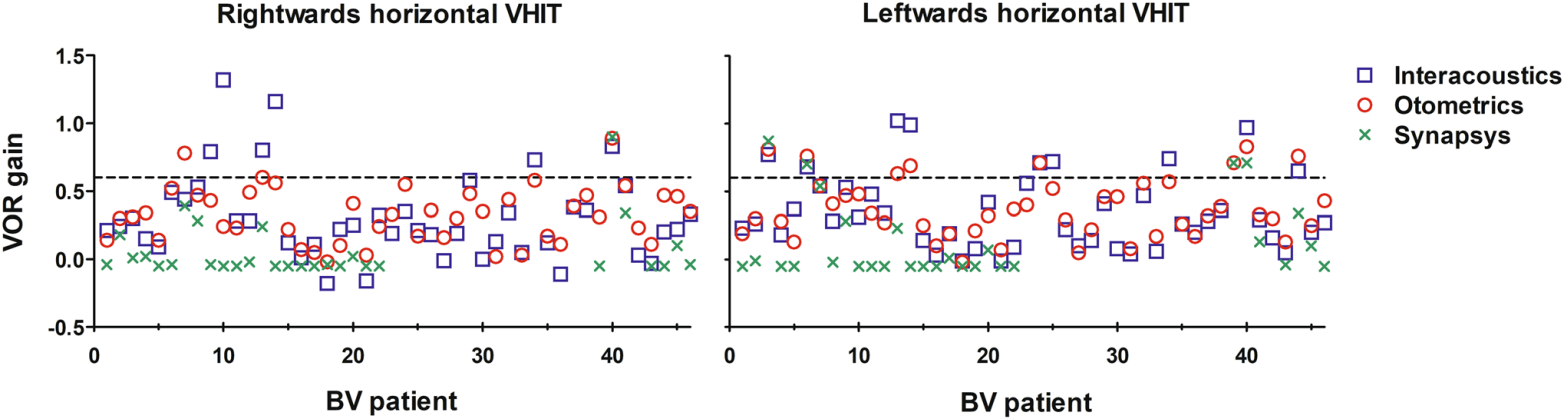
VOR gains for leftwards and rightwards horizontal VHIT, as tested with three different VHIT systems. Every symbol represents the VOR gain of one VHIT trial in one patient obtained with one VHIT system. The horizontal line at a VOR gain of 0.6 represents the cut-off value according to the BV criteria of the Bárány society [8]. VOR gains obtained with the Synapsys system differed significantly from VOR gains obtained with the other two systems. No statistically significant difference was found in VOR gains between the Interacoustics and Otometrics system

Figure 3 illustrates that different VOR gains were obtained by different VHIT systems, within the same BV patients. There was a statistically significant difference between the three systems in VOR gains [*F*(1.256, 33.916) = 35.681, *p* < 0.000]. VOR gains obtained with the Synapsys system differed significantly from VOR gains obtained with the other two systems. No statistically significant difference was found in VOR gains between the Interacoustics and Otometrics system. Mean VOR gains of all patients were 0.33, 0.35 and 0.10 for Interacoustics, Otometrics and Synapsys system, respectively.

The VHIT systems agreed in 83% of the 46 patients on the BV diagnosis (“bilateral vestibulopathy” or “no bilateral vestibulopathy”) according to the criteria of the Bárány Society [8]. In eight patients (17%), no agreement was found (Table 1). These eight patients were diagnosed with BV resulting from various etiologies: ototoxic effects of gentamicin (*n* = 1) and chemotherapy (*n* = 1), bilateral Menière’s disease (*n* = 1), post-infectious due to Lyme’s disease, (*n* = 1) inherited (*n* = 1), and idiopathic (*n* = 3).

**Table 1:** Differences between VHIT systems, when diagnosing BV only based on VOR gains

Diagnosis according to VHIT results	Interacoustics (*N* = 45)	Otometrics (*N* = 46)	Synapsys (*N* = 28)	All patients (*N* = 46)
Bilateral vestibulopathy	76%	80%	86%	72%
No bilateral vestibulopathy	24%	20%	14%	11%
No agreement between systems	16% Otometrics 24% Synapsys	16% Interacoustics 17% Synapsys	24% Interacoustics 17% Otometrics	17%

Horizontal VOR gain of < 0.6 was classified as “bilateral vestibulopathy”, a VOR gain of ≥ 0.6 was classified as “no bilateral vestibulopathy”. In case VHIT systems showed a discrepancy in diagnosis of BV, the patient was classified as “no agreement”. Not all patients were tested with all three systems since systems were not always available at time of testing

In the 28 patients tested with all three VHIT systems, the percentage of agreement between the VHIT systems was 79% (68% BV, 11% no BV), and in 21% there was no agreement. The mean VOR gains obtained in these 28 patients were 0.36, 0.36 and 0.09 for Interacoustics, Otometrics and Synapsys respectively.

### VOR gain and repetitive testing

No order effect was present, since no difference in VOR gain was found between the first and the last VHIT trials, regardless of the system used for VHIT.

### Peak head velocity

For every VHIT system, median peak head velocities with their interquartile range of all traces together from all patients are presented in Table 2. A significant difference in the velocity of the head impulses between the three systems was found (*p* < 0.001). Regarding the Synapsys system, significantly lower median peak head velocities (maximum 43°/s lower) and VOR gains (maximum 0.37 lower) were present than in the other two systems. Interacoustics and Otometrics did not significantly differ regarding VOR gain, only regarding median peak head velocity (maximum 11°/s).

**Table 2:** Median peak head velocities [with their first (Q1) and third quartiles (Q3)] and median VOR gain (as calculated by the VHIT system) for rightwards and leftwards horizontal head impulses

	Rightwards horizontal VHIT			Leftwards horizontal VHIT		
VHIT system	Peak head velocity	Q1|Q3	VOR gain	Peak head velocity	Q1|Q3	VOR gain
Interacoustics	207	183|229	0.22	198	175|217	0.28
Otometrics	215	192|240	0.32	209	186|231	0.33
Synapsys	178	156|200	− 0.04	166	135|195	− 0.04

There was a statistically significant difference in peak head velocities between the three systems. Both peak head velocity and VOR gain were lower in Synapsys than in the other two systems

Peak head velocities were separately analysed in the eight patients with “no agreement” on the diagnosis of BV according to the VHIT systems (Fig. 3). In one out of the eight patients, the median peak head velocity of the given head impulses was significantly higher in the system with the lower VOR gain. This patient showed in the Interacoustics system a VOR gain of 0.74 with median peak head velocity of 196°/s (leftwards impulses) and a VOR gain of 0.73 with median peak head velocity of 214°/s (rightwards impulses), versus a VOR gain of 0.57 with median peak head velocity of 265°/s (leftwards impulses) and a VOR gain of 0.58 with median peak head velocity of 255°/s (rightwards impulses) in the Otometrics system.

In the other seven patients, no statistically significant difference in peak head velocities between VHIT systems was found, or the system with significantly higher (or lower) peak head velocities also measured higher (or lower, respectively) VOR gains in that patient [19].

### Frequency and latency of covert saccades

**Fig. 4:**
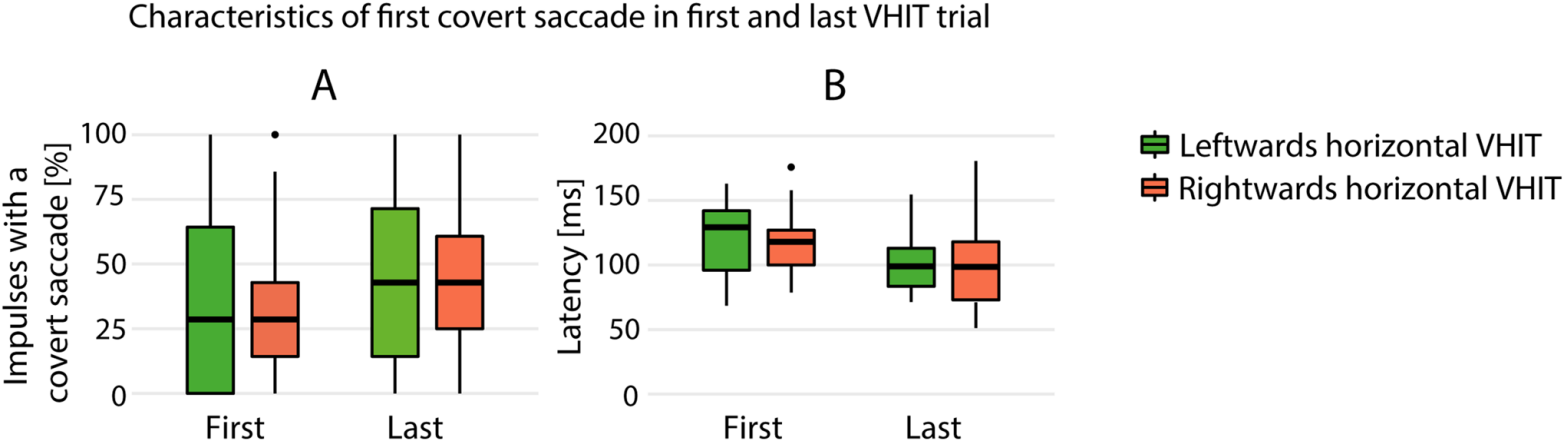
Characteristics of the first appearing covert saccade from the first seven artefact-free traces of all patients together. **a** The frequency of covert saccades (percentage of impulses with at least one covert saccade) in the first and last VHIT trial. **b** Latency of the first covert saccade (the moment of onset of the saccade in milliseconds, start of head impulse is 0 ms) in the first and last VHIT trials. No statistically significant difference was found within the characteristics between the first and the last VHIT trials regardless of the VHIT system (Interacoustics, Otometrics or Synapsys)

According to the strict methods as described above, frequency of covert saccades could be analysed in 34 patients, and latency of covert saccades in 20 patients. In this study, no statistically significant difference in the frequency of occurrence of covert saccades and in the latency of the first appearing covert saccade was found between the first and the last VHIT trials, regardless of the system (Fig. 4).

## Discussion

This study compared the VOR gains obtained with three commercially available VHIT systems (Interacoustics, Otometrics and Synapsys) in a large group of BV patients. In 83% of the patients the VHIT systems agreed on the diagnoses of BV, when using a cut-off horizontal VOR gain of < 0.6 [8]. Additionally, while VOR gains did not significantly differ between the Interacoustics and Otometrics system, they both significantly differed from VOR gains obtained with the Synapsys system.

The fact that agreement between VHIT systems on BV diagnosis was present in 83% of the cases implies that in 17%, no agreement was present. This is suboptimal for diagnostic devices used in clinical setting. It would be preferred to further investigate the origin of these differences in outcome between VHIT systems, to improve the diagnostic pathway in BV patients. The origin might have (partially) resulted from inherent differences in the VHIT systems themselves, e.g. differences in eye and head tracking, and/or VOR gain calculation. This has been described before in healthy subjects, but this is the first study that shows the possible significant impact on the diagnosis of BV [5, 6]. It has been hypothesised that mainly the differences in VOR gain calculation algorithm are responsible for the VOR gain differences (van der Lans, manuscript in preparation). After all, especially in BV patients, the transfer function of the VOR is often not linear, and the appearance of covert saccades might interfere with VOR gain calculation. This implies that VOR gain outcomes are very sensitive to pre-processing (e.g. desaccading) and interpretation of the traces by the VOR gain calculation algorithm. To overcome some of these challenges, the Suppression Head Impulse Test (SHIMP) was proposed, that might decrease the amount of covert saccades and better show the residual vestibular function [3, 13, 20]. However, this paradigm still depends on the VOR gain calculation algorithm, and its clinical relevance in BV is yet to be determined (van Dooren, manuscript in preparation). Generally, it seems therefore necessary that VHIT systems are standardised regarding eye and head tracking methods and VOR gain calculation algorithms, to improve proper diagnosis of BV. If this is not possible, it could be investigated whether VHIT system-specific cut-off values to diagnose BV are a possibility to increase agreement between VHIT systems. Nevertheless, it remains important to not only assess VOR gain, but also the raw traces and compensatory saccades. In addition, BV is diagnosed using a combination of symptoms and several vestibular tests (caloric test, rotatory chair test, VHIT). Since these vestibular tests are complementary, only performing VHIT might not be enough to rule out BV [8, 21].

In this BV population, outcomes of the Synapsys system differed significantly from the other two VHIT systems: Synapsys showed a lower VOR gain than Interacoustics and Otometrics (Fig. 3). This could (partially) be explained by differences in gain calculation algorithms, different eye- and head tracking methods (Synapsys uses a space-fixed camera, the other two systems use a camera fixed to a pair of goggles), or differences in sampling frequency (Synapsys uses a lower sampling frequency of 100 Hz, compared to 220 Hz and 245 Hz for Interacoustics and Otometrics, respectively). Furthermore, during visual inspection the Synapsys system showed less smooth eye velocity traces, and more missing values than Interacoustics and Otometrics (Fig. 2). However, when the Synapsys system considered a patient “no BV” (VOR gain ≥ 0.6), this was always in agreement with both of the other two systems. Nevertheless, the other way around (“BV” with Synapsys and “no BV” in the other two systems) also occurred. It is unknown whether this was a systematic mistake of the Synapsys system, or whether Synapsys was the only system that was able to best detect BV in the high-frequency range of this population. This question was beyond the scope of this article, but could be addressed in the future.

When observing differences in VOR gains between different VHIT systems and VHIT trials, it is very important to first rule out measurement artefacts, like clinically relevant differences in peak head velocities, the order effect, and differences in frequency and latency of covert saccades that could influence the VOR gain calculations [5, 16, 21]. Regarding differences in peak head velocities, a higher peak head velocity might result in lower VOR [19]. However, in contrast to these findings, the system with significantly lower median peak head velocities during VHIT trials (Synapsys), also showed the lowest VOR gains in this study. Therefore, it is very unlikely that differences in peak head velocity between Synapsys and the other VHIT systems might have caused most of the VOR gain differences between VHIT systems in this study. The statistically significant difference in median peak head velocities between VHIT trials of Interacoustics and Otometrics was only small (11°/s difference), and, therefore, probably not influenced the (not significant) VOR gain differences between the two systems [19]. Regarding the order effect and the frequency and latency of covert saccades, VOR gains and covert saccades did not show differences in this BV population with repetitive testing. This is in agreement with previous studies on healthy subjects and patients with vestibular dysfunction [7, 22]. Therefore, it can be concluded that it is very unlikely that measurement artefacts like the order effect or covert saccades could explain the significant differences in VOR gains found between the three VHIT systems in this study.

### Limitations

In patients with low VOR gains, biphasic eye movement artefacts can occur at the beginning of head impulses, when using a head-mounted VHIT system (e.g. Fig. 2, eye movements obtained during rightward impulses with Interacoustics and Otometrics system). This might lead to erroneous higher VOR gains, especially when using the instantaneous gain calculation method (Interacoustics) compared to the area under the curve gain calculation method (Otometrics) [13, 16]. This type of artefact was not specifically addressed in this study. Since VOR gains obtained with the Interacoustics and Otometrics systems did not significantly differ in this study, comparison of these two systems was most likely not compromised by this artefact. However, it cannot be ruled out that this artefact might (partially) explain some of the relatively lower VOR gains in the Synapsys system.

## Conclusion

To conclude, using different VHIT systems in the same BV patient can lead to clinically significant differences in VOR gain, when using a cut-off value of 0.6. This might hinder proper diagnosis of BV patients. It would, therefore, be preferred that VHIT systems are standardised regarding eye and head tracking methods, and VOR gain calculation algorithms. Until then, it is advised to not only take the VOR gain in consideration when assessing a VHIT trial, but also look at the raw traces and the compensatory saccades.

## Data Availability

The data that support the findings of this study are available from the corresponding author, TD, upon reasonable request.
